# Ultrasound features of pregnancy‐associated breast cancer: A retrospective observational analysis

**DOI:** 10.1002/cam4.4974

**Published:** 2022-06-23

**Authors:** Maryam Jafari, Fereshteh Abbasvandi, Elahe Nazeri, Asiie Olfatbakhsh, Ahmad Kaviani, Rezvan Esmaeili

**Affiliations:** ^1^ Department of Radiology Ali Asghar Children Hospital, Iran University of Medical Sciences Tehran Iran; ^2^ Genetics Department, Breast Cancer Research Center Motamed Cancer Institute, ACECR Tehran Iran; ^3^ ATMP Department, Breast Cancer Research Center Motamed Cancer Institute, ACECR Tehran Iran; ^4^ Breast Diseases Department, Breast Cancer Research Center Motamed Cancer Institute, ACECR Tehran Iran; ^5^ Department of Surgery Tehran University of Medical Science Tehran Iran

**Keywords:** BI‐RADS, non‐PABC, pathological characteristics, pregnancy‐associated breast cancer, ultrasound

## Abstract

Pregnancy‐associated breast cancer (PABC) is a poor prognosis in women, and the mortality rate is higher in this subgroup of patients than in non‐PABC. This study aims to assess clinicopathological and ultrasound features of patients with PABC. Of 75 patients with breast cancer, 31 cases were in lactating, or pregnancy phase and 44 patients had no recent history of pregnancy/lactation at the time of cancer detection. The available pathological characteristics and ultrasound findings of the PABC and non‐PABC groups were compared. The analysis of ultrasound findings demonstrated that the percentages of antiparallel orientation (*p* = 0.04) and heterogeneous internal echo pattern (*p* = 0.002) were higher in the PABC group. The final Breast Imaging Reporting and Data System (BI‐RADS) assessment in the two groups was significantly different (*p* = 0.008). In this study, most PABCs were BI‐RADS 4c or 5; compared with age‐matched non‐PABC cases. There were significant differences in ER (*p* = 0.03), receptor groups (*p* = 0.007), and tumor grade (*p* = 0.02) in PABC compared to non‐PABC group. To conclude, radiologists should be careful about ultrasound findings of PABC and recommend core needle biopsy in suspected cases.

## Graphical abstract

Diagnosis of breast cancer during pregnancy and lactation is commonly delayed, mostly due to the difficulty of tumor detection in thick fibroglandular tissue and low awareness among patients. This study evaluates ultrasound findings of PABC and provides an approach for the assessment of pregnant and lactating patients, especially with palpable disease or thickening sensation. Familiarity of breast imaging radiologists with multi‐modality features of PABC is essential to early diagnosis.

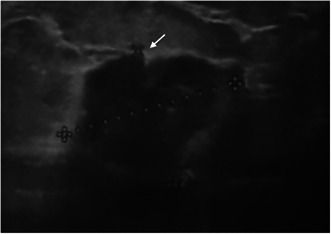

## 1 INTRODUCTION

Breast cancer puts a heavy burden on health systems around the world. In developing countries, such as Iran, women's age of breast cancer onset is lower and so many patients are of childbearing ages. Therefore, the relative frequency of breast cancer cases in pregnancy and lactation is higher. Pregnancy‐associated breast cancer (PABC) occurs during pregnancy, in the first post‐partum year, or during lactation. However, some authors have expanded this period up to 2 years during lactation. PABC has an incidence of 0.3 per 1000 pregnancies.^1^, ^2^, ^3^, ^4^

On the other hand, these patients are less likely to be diagnosed due to the lack of attention to breast diseases during this period. As a result, early diagnosis and early treatment are critical as these cancers generally have a worse prognosis, and they are often associated with axillary metastasis and larger tumors.^5^

Nowadays, childbearing is delayed until the 30th or 40th decade which is associated with an increased risk of PABC. Most PABC patients are diagnosed in the first 6 months following delivery. The PABC tumors are larger and more advanced than age‐matched non‐PABC cases, and patients usually have a palpable abnormality in the physical exam.^6^, ^7^ Whole breast ultrasound is commonly used as the initial modality for detecting PABC. Herein, ultrasound features of PABC were evaluated with aged‐matched non‐PABC cases; emphasizing that mammographic and sonographic features of the PABC tumors may differ from non‐PABC ones as declared in a few prior study.^7^ They may be due to physiologic and hormonal changes in pregnancy and lactating.

Mammography and ultrasound are essential to detect abnormalities and microcalcifications in a diagnostic setting. Ultrasonography has high sensitivity and specificity in PABC and is the initial step in dealing with pregnant and breastfeeding patients with mass sensation. Since breast nodularity increases in pregnancy and lactation period, any stable nodularity that lasts more than 2–4 weeks requires an ultrasound. In some studies, the sensitivity of ultrasound has been reported up to 100%, with a negative predictive value of about 100%.^8^, ^9^

Based on sonographic Breast Imaging Reporting and Data System (BI‐RADS), suspicious appearing descriptors such as spiculated margin, irregularity, and non‐parallel orientation have high predictive value for malignant tumors. In contrast, circumscribed wall, oval/gently lobulated shape, and parallel orientation are in favor of benign masses. However, there are physiological changes in lactating and pregnancy phases that may change these typical features of malignancy in this phase.^10^ Parallel orientation and posterior enhancement have been reported in 58% and 63% of PABC patients, respectively.^10^ The most common ultrasound feature of PABC is an irregular‐shaped hypoechoic indistinct mass. In the pregnancy phase, physiological thicken fibroglandular tissues are developed. The ductal system is prominent in the lactating phase; while breast vascularity increases in both phases.^7^, ^11^, ^12^, ^13^, ^14^ After cessation of lactation, the ultrasound features of the cancers return to the pre‐pregnancy state.^15^ In another study, 30% of the tumors with posterior enhancement had large cystic components common due to central necrosis.^13^, ^16^

Diagnosis of breast cancer is commonly delayed during pregnancy and lactation, mostly due to the difficulty of tumor detection in thick fibroglandular tissue and low awareness among patients. Familiarity with breast imaging radiologists with multi‐modality features of PABC is essential for early diagnosis. This article evaluates ultrasound findings of PABC and provides an approach for the assessment of pregnant and lactating patients, especially those with palpable disease or thickening sensation.

## 2 MATERIALS AND METHODS

### 2.1 Study population

From November 2015 to December 2021, 2400 patients were diagnosed with breast cancer at Motamed cancer institute. Of these, 31 met our inclusion criteria as a PABC allocated to the case group. Our inclusion criteria for the case group allowed only for patients whose breast cancer was diagnosed during pregnancy or in the first and second post‐partum year or during lactation. The control group (non‐PABC) included 44 age‐matched patients who had no recent history of pregnancy/lactation at the time of cancer detection. For the case group, we chose a control group in the same six‐year age group. Since PABC is a rare disease, we chose more controls to avoid patient miss selection. The mean age of diagnosis in the case group was 35.41 (24–42) years and 36.75 (30–40) years in the control group.

### 2.2 Clinical and pathological data collection

Clinical and pathological records of all women with breast cancer who had not undergone surgery, neoadjuvant chemotherapy, or any treatment related to their cancer referred to our institution were collected and assessed.

The pathological characteristics of the breast tumors, including estrogen, progesterone, HER‐2/neu receptor status, tumor grade, and lymph node were determined in both groups. All the patients' pathology reports were invasive ductal carcinoma.

### 2.3 Ultrasound appearance

Two expert breast imaging radiologists with 8–10 years of experience reviewed all available sonography records. Breast masses were categorized based on BI‐RADS. Sonograms were assessed to reveal breast composition, presence of masses, and their features; including shape, margins, orientation, echo pattern, posterior features, and the presence of calcifications. Also, both case and control groups were studied for associated features such as distortion, retraction, lymphadenopathy, and the cyst's existence. Final BI‐RADS assessment categories were assigned based on these descriptors. We decided to compare and divide BI‐RADS of lesions suspected of malignancy into subgroups; including the low to intermediate‐risk cases (under 50%, BI‐RADS 4a or 4b) and high‐risk cases (over 50%, BI‐RADS 4c or 5).

### 2.4 Statistical analysis

The determined data of both studied groups; including clinical characteristics and imaging results, were analyzed with the Chi‐square test. *p*‐value <0.05 was considered for statistically significant. IBM SPSS statistics for windows, version 23.0 (IBM Corp) was used for descriptive statistical analysis.

## 3 RESULTS

### 3.1 Pathological information

The clinical characteristics of both groups are summarized in Table 1. Accordingly, there was a significant difference in ER receptor (*p* = 0.03) and receptor groups (*p* = 0.007). Furthermore, there was a significant difference in pathological grades (*p* = 0.02) in PABC compared to the non‐PABC group.

**TABLE 1:** Clinical characteristics of the patients in the case and control group

Case/control groups		Case	Control	*p* value
Number		31	44	
Age mean (range) — year		35.41 (24–42)	36.75 (30–40)	0.17
ER status (1 missing, *n* = 75)	Negative	14 (46.7%)	10 (22.7%)	**0.03**
	Positive	16 (53.3%)	34 (77.3%)	
PR status (1 missing, *n* = 75)	Negative	15 (50%)	13 (29.5%)	0.07
	Positive	15 (50%)	31 (70.5%)	
HER2/neu status (2 missings, *n* = 75)	Negative	21 (72.4%)	27 (61.4%)	0.3
	Positive	8 (27.6%)	17 (38.6%)	
Receptor groups (2 missings, *n* = 75)	ER + PR ± Her2−	17 (58.8%)	25 (56.8%)	**0.007**
	Her2+	4 (13.8%)	17 (38.6%)	
	ER/PR−, Her2−	8 (27.6%)	2 (4.5%)	
Tumor Grade (5 missings, *n* = 75)	G1	2 (7.1%)	4 (9.5%)	**0.02**
	G2	21 (75%)	18 (42.9%)	
	G3	5 (17.9%)	20 (47.6%)	

### 3.2 Ultrasound findings

All lesions were mass and were classified according to the fifth ultrasound BI‐RADS lexicon. The data relating to both groups are summarized in Table 2. The analysis demonstrated that the percentage of an antiparallel orientation (*p* = 0.04) and heterogeneous internal echo pattern (*p* = 0.002) were higher in the case group. There were no significant differences in other variables.

**TABLE 2:** Ultrasound features of the patients in the case and control group

				Case	Control	*p* value
				*N*	*N*	
				31	44	
Masses	Shape (6 missings, *n* = 75)	Oval		3 (10%)	10 (25.6%)	0.2
		Round		2 (6.7%)	2 (5.1%)	
		Irregular		25 (83.3%)	27 (69.2%)	
	Orientation (7 missings, *n* = 75)	Parallel		3 (9.7%)	11 (25%)	**0.04**
		Non‐parallel		27 (87.1%)	27 (61.4%)	
	Margin (6 missings, *n* = 75)	Circumscribed		3 (10%)	1 (2.6%)	0.2
		Non‐Circumscribed	Angular	2 (6.7%)	1 (2.6%)	
			Well‐defined	1 (3.3%)	0	
			Microlobulated	3 (10%)	10 (25.6%)	
			Indistinct	5 (16.7%)	10 (25.6%)	
			Spiculated	16 (53.3%)	17 (43.6%)	
	Internal Echo (7 missings, *n* = 75)	Homogeneous hypo		3 (10.3%)	19 (48.7%)	**0.002**
		Heterogeneous		26 (89.7%)	19 (48.7%)	
		Complex cystic		0 (0%)	1 (2.6%)	
	posterior features (5 missings, *n* = 75)	No posterior features		9 (29%)	12 (30.8%)	0.8
		Enhancement		2 (6.5%)	2 (5.1%)	
		Shadowing		16 (51.6%)	22 (56.4%)	
		Combined pattern		4 (12.9%)	3 (7.7%)	
Cystic	Yes			3 (9.7%)	0	0.07
	No			28 (90.3%)	44 (100%)	
BI‐RADS (6 missings, *n* = 75)	4a, 4b			6 (19.4%)	19 (50%)	**0.008**
	4c, 5			25 (80.6%)	19 (50%)	

**FIGURE 1:**
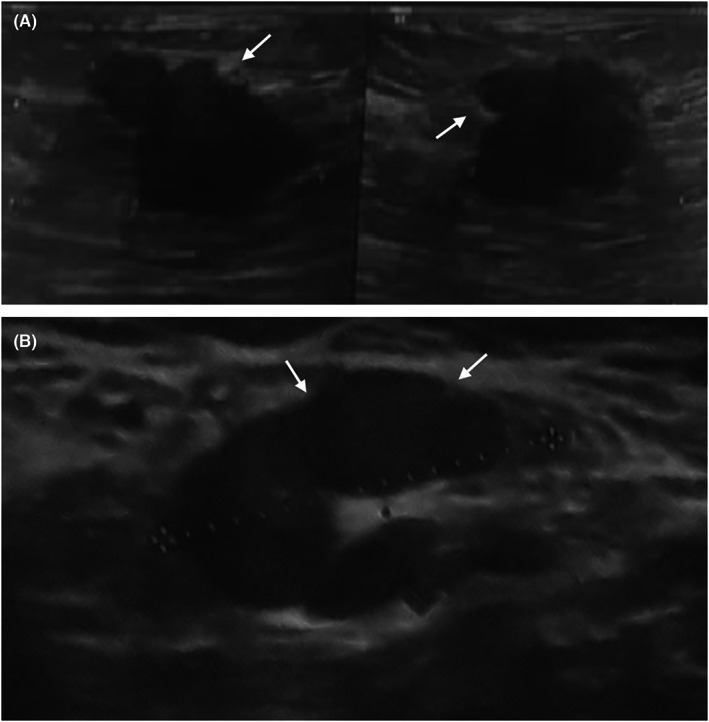
(A): A 37‐year‐old lactating woman, presented with a palpable mass with irregular shaped, microlobulated (arrowhead) margin and taller than wide orientation in ultrasound 2 months post‐partum. ER‐negative and HER2‐negative invasive ductal carcinoma was proved by Ultrasound‐guided biopsy. (B): A 37‐year‐old lactating woman with a suspicious appearing lymph node revealed a metastasis from triple‐negative invasive ductal carcinoma with ultrasound‐guided biopsy 2 months post‐partum.

Figure 1A is the ultrasound image of a 37‐year‐old lactating woman with a palpable mass; presented 2 months after delivery. The mass had an irregular shape, microlobulated margin, and was taller than wide in ultrasound orientation. Ultrasound‐guided biopsy proved ER‐negative and HER2‐negative invasive ductal carcinoma. In Figure 1B, a 37‐year‐old lactating woman with a suspicious appearing lymph node, presented in the second post‐partum month, revealed a metastasis from triple‐negative invasive ductal carcinoma after ultrasound‐guided biopsy.

**FIGURE 2:**
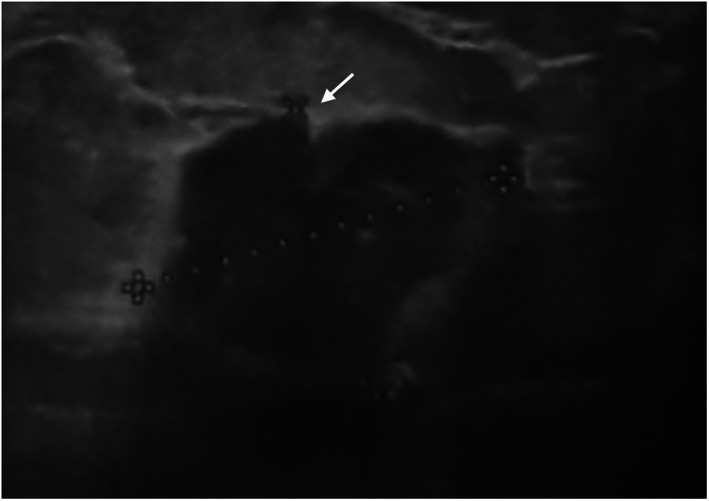
32‐year‐old pregnant woman with suspicious irregular angular/ spiculated mass 26 weeks after gestation.

In Figure 2, a 32‐year‐old pregnant woman was presented with a suspicious irregular spiculated mass in the 26th gestational week. Ultrasound‐guided biopsy revealed ER/PR‐positive and HER 2‐negative invasive ductal carcinoma.

### 3.3 Sonographic BI‐RA DS classification

The final BI‐RADS assessment was significantly different (*p* = 0.008) in these two groups. Most of the PABC groups were BI‐RADS 4c or 5; while in the non‐PABCs, the distribution of the mass BI‐RADS was similar (Table 2).

## 4 DISCUSSION

According to studies among the diagnostic modalities for PABC, ultrasound is the best method to examine suspicious breast masses during pregnancy. Still, there is no study to compare the ultrasound findings of PABC and non‐PABC patients. In this regard, the present study may add valuable information to the current knowledge in this area of cancer science.

In our research, most PABCs were masses with spiculated or indistinct margins in ultrasound examinations and showed irregular‐shaped and non‐parallel orientation. Considering mass shape, this was compatible with the previous study of Taylor et al. in which the most common ultrasound feature was irregularity.^17^ Some ultrasound descriptors including internal echogenicity of the mass and mass orientation were significantly different; comparing PABCs and non‐PABCs. The PABCs were mostly heterogeneous and non‐parallel in 89.7% and 87.1% of cases, respectively which is a new finding compared to previous studies. The heterogenicity may be due to the high growth rate of the mass and their pathological nature. The rate of ER‐ patients in the case group is higher than in controls. Moreover, triple‐negative tumors were more common in the case group. In contrast, the control group had more Her2‐positive tumors than the case. The low number of cases due to the low prevalence of PABC should be considered when interpreting the results.

In this study, all of the cases had BI‐RADS 4 or BI‐RADS 5; while it was 77% in the study of Langer et al.^18^ These differences may be due to the limited number of cases in the present study.

Unfortunately, limited studies exist in the literature, considering the imaging features of PABC. In contrast to the prior research of Ahn et al., only three of the PABC masses had a cystic component (9.7%), and two of the PABC masses had a posterior enhancement (6.5%) in the present study.^16^

Mammography should be suggested to detect occult malignant microcalcifications. If a focal palpable lesion is not seen with imaging modalities, a palpable mass or thickening sensation should be biopsied in pregnant patients or further evaluated by MRI/or biopsy in lactating phase.^10^, ^19^

This retrospective study has limitations such as small sample size, not using Doppler or elastography, and mammography findings. Therefore, prospective studies are needed to examine this critical issue in a larger patient group in which all diagnostic aspects of the imaging modalities have been considered.

## 5 CONCLUSION

In conclusion, the incidence of PABC would increase because of delays in childbearing until the third and fourth decades of life. The initial imaging of choice for the breast assessment, especially in symptomatic cases, is high‐resolution ultrasound. The suspicious masses are the most prevalent shape of the mass in the ultrasound. In this study, all the PABCs showed BI‐RADS 4 or BI‐RADS 5, and most of them have BI‐RADS 4c or BI‐RADS 5 as compared with the age‐matched non‐PABCs. Furthermore, there were significant differences in ER, receptor groups, and tumor grade in PABCs compared to the non‐PABC group. Radiologists should be aware of ultrasound findings of PABC patients and warrant core needle biopsy in suspected cases. As the experience in PABC is limited regarding the low number of reported cases, caution should be considered in drawing any conclusions.

## AUTHORS CONTRIBUTIONS

Maryam Jafari: Conceptualization, radiographic data extraction, drafting the manuscript.

Fereshteh Abbasvandi: Diagnosis and referring patients.

Elahe Nazeri: Collecting samples and drafting the manuscript.

Asiie Olfatbakhsh: Providing control group data.

Ahmad Kaviani: Diagnosis, referring patients, and revising the article.

Rezvan Esmaeili: Conceptualization and designing the project, providing fund and data analysis, critically revising the article, and approving the final version for publication.

All authors read and approved the final manuscript.

## ETHICS STATEMENT

The Ethical Committee of Motamed Cancer Institute approved the study. The ethics code is IR.ACECR.IBCRC.REC.1398.002. Informed consent was obtained from all patients.

## Funding information

This project was supported by Iran National Science Foundation (INSF), (Grant Number: 95849123) and The Academic Center for Education, Culture and Research (ACECR), (Grant Number: 3084–20).

## CONFLICT OF INTEREST

The authors declare that there is no conflict of interest.

## Data Availability

The data that support the findings of this study are available from the corresponding author upon request.
